# Supplementary material for: Regulation of mRNA translation by a photoriboswitch
Source: eLife. 2020 Feb 13;9:e51737. doi: 10.7554/eLife.51737 (PMC7051177; doi:10.7554/eLife.51737)
Supplement: Supplementary file 1. [file elife-51737-supp1.docx]

**Supplementary File 1**

**Supplementary Table 1**

**Regulation of mRNA translation by a photoriboswitch**

Kelly A. Rotstan^[[1]](#footnote-1)^†, Michael M. Abdelsayed^[[2]](#footnote-2)^†, Luiz F. M. Passalacqua^1^, Fabio Chizzolini^1^, Kasireddy Sudarshan^4^, A. Richard Chamberlin^1,^^[[3]](#footnote-3)^, Jiří Míšek^1,^^[[4]](#footnote-4)*^ & Andrej Lupták^1,2,3*^

**Supplementary Table 1.**

**DNA sequences used for *in vitro* and *in vivo* analysis of the Were-1** **riboswitch.**

| Toehold-Fluorophore Reporter | Rep F: 5'– 5FluorT/TA CCTGCAAGCTTCCCTTTTCAAAATAAAAACCCCT –3'  Rep Q: 5'– ATTTTGAAAAGGGAAGCTTGCAGGTA/3IABkFQ –3' |
| --- | --- |
| Forward Overhang Primer (AL2909) | 5'– CCCgaattcTAATACGACTCACTATAGGGATCCGAAAATTGAAATAGAC –3' |
| Reverse Overhang Primer (AL2912) | 5'– atggcgccgggcctttctttatgtttttggcgtcttCCCTGCAAGCTTCCCTTTTCAAAATAAAAAC –3' |
| Forward Werewolf Primer (AL2881) | 5'– GGGATCCGAAAATTGAAATAGACTCC –3' |
| Reverse Nested Fluc Primer (AL1649) | 5'– gattctgtgatttgtattcagcccata –3' |
| Pool Forward Primer (AL2045) | 5'– TAATACGACTCACTATAGGGATCCGAAAATTGAAATAGAC –3' |
| Pool Reverse Primer (AL2049) | 5'– TACCTGCAAGCTTCCCTTTTC –3' |
| AUG mutant Forward Primer (AL3267) | 5'– 5Phos/ATGAAAAGGGAAGCTTGCAGGgaagac –3' |
| AUG mutant Reverse Primer (AL3268) | 5'– /5Phos/AA TAA AAA CCC CTT CTT CAA GGT TGG CTG AAG –3' |
| C89G mutant Forward Primer (AL3330) | 5'– /5Phos/GACATCTTCAGCCAACCTTG – 3’ |
| C89G mutant Reverse Primer (AL3331) | 5'– /5Phos/GTTTGATGCTTCTGGTCATCTG –3' |
| G69C mutant Forward Primer (AL3328) | 5'– /5Phos/CATGACCAGAAGCATCAAACCAC –3' |
| G69C mutant Reverse Primer (AL3329) | 5'– /5Phos/TGGTAATTCACGGTGCTTACTGC –3' |
| C91G mutant Forward Primer  (AL3326) | 5'– /5Phos/GATCTTCAGCCAACCTTGAAGAAGG –3' |
| C91G mutant Reverse Primer  (AL3327) | 5'– /5Phos/TGGTTTGATGCTTCTGGTCATCTG –3' |
| C84G/G69C compensatory mutant Forward Primer (AL3384) | 5'– /5Phos/GAAACCACATCTTCAGCCAACCTT–3' |
| C84G/G69C compensatory mutant Reverse Primer (AL3385) | 5'– /5Phos/ATGCTTCTGGTCATGTGGTAATTCAC –3' |
| Were-1 DNA | 5'–GGGATCCGAAAATTGAAATAGACTCCGATGGACGGTTGCGTGTATGCAGTAAGCACCGTGAATTACCAGATGACCAGAAGCATCAAACCACATCTTCAGCCAACCTTGAAGAAGGGGTTTTTATTTTGAAAAGGGAAGCTTGCAGGTA –3' |
| Amino-*t*SS pool | 5'–TAATACGACTCACTATAGGGATCCGAAAATTGAAATAGAC111111111111111111111111111111111111111111111452554554554342255533332232231111111111GAAGAAGGGGTTTTTATTTTGAAAAGGGAAGCTTGCAGGTA–3' |
| Were-1-Fluc construct | 5'–TAATACGACTCACTATAGGGATCCGAAAATTGAAATAGACTCCGATGGACGGTTGCGTGTATGCAGTAAGCACCGTGAATTACCAGATGACCAGAAGCATCAAACCACATCTTCAGCCAACCTTGAAGAAGGGGTTTTTATTTTGAAAAGGGAAGCTTGCAGGgaagacgccaaaaacataaagaaaggcccggcgccattctatccgctggaagatggaaccgctggagagcaactgcataaggctatgaagagatacgccctggttcctggaacaattgcttttacagatgcacatatcgaggtggacatcacttacgctgagtacttcgaaatgtccgttcggttggcagaagctatgaaacgatatgggctgaatacaaatcacagaatcgtcgtatgcagtgaaaactctcttcaattctttatgccggtgttgggcgcgttatttatcggagttgcagttgcgcccgcgaacgacatttataatgaacgtgaattgctcaacagtatgggcatttcgcagcctaccgtggtgttcgtttccaaaaaggggttgcaaaaaattttgaacgtgcaaaaaaagctcccaatcatccaaaaaattattatcatggattctaaaacggattaccagggatttcagtcgatgtacacgttcgtcacatctcatctacctcccggttttaatgaatacgattttgtgccagagtccttcgatagggacaagacaattgcactgatcatgaactcctctggatctactggtctgcctaaaggtgtcgctctgcctcatagaactgcctgcgtgagattctcgcatgccagagatcctatttttggcaatcaaatcattccggatactgcgattttaagtgttgttccattccatcacggttttggaatgtttactacactcggatatttgatatgtggatttcgagtcgtcttaatgtatagatttgaagaagagctgtttctgaggagccttcaggattacaagattcaaagtgcgctgctggtgccaaccctattctccttcttcgccaaaagcactctgattgacaaatacgatttatctaatttacacgaaattgcttctggtggcgctcccctctctaaggaagtcggggaagcggttgccaagaggttccatctgccaggtatcaggcaaggatatgggctcactgagactacatcagctattctgattacacccgagggggatgataaaccgggcgcggtcggtaaagttgttccattttttgaagcgaaggttgtggatctggataccgggaaaacgctgggcgttaatcaaagaggcgaactgtgtgtgagaggtcctatgattatgtccggttatgtaaacaatccggaagcgaccaacgccttgattgacaaggatggatggctacattctggagacatagcttactgggacgaagacgaacacttcttcatcgttgaccgcctgaagtctctgattaagtacaaaggctatcaggtggctcccgctgaattggaatccatcttgctccaacaccccaacatcttcgacgcaggtgtcgcaggtcttcccgacgatgacgccggtgaacttcccgccgccgttgttgttttggagcacggaaagacgatgacggaaaaagagatcgtggattacgtcgccagtcaagtaacaaccgcgaaaaagttgcgcggaggagttgtgtttgtggacgaagtaccgaaaggtcttaccggaaaactcgacgcaagaaaaatcagagagatcctcataaaggccaagaagggcggaaagatcgccgtgtaa–3' |

**Abbreviations/Key**

3IABkFQ – 3' Iowa black fluorophore quencher

5FluorT – 5' Fluorescein

1 A:G:C:T=1:1:1:1

2 A:G:C:T=17:1:1:1

3 A:G:C:T=1:17:1:1

4 A:G:C:T=1:1:17:1

5 A:G:C:T=1:1:1:17

1. Department of Pharmaceutical Sciences, University of California, Irvine, 92697, USA. [↑](#footnote-ref-1)
2. Department of Molecular Biology and Biochemistry, University of California, Irvine, 92697, USA. [↑](#footnote-ref-2)
3. Department of Chemistry, University of California, Irvine, 92697, USA. [↑](#footnote-ref-3)
4. Department of Organic Chemistry, Charles University, Prague, Czech Republic.

   †These authors contributed equally to this work. [↑](#footnote-ref-4)
